# The Field and Limitation of the Operative Surgery of the Brain

**Published:** 1886-08

**Authors:** 


THE FIELD AND LIMITATION OF THE OPERATIVE SURGERY OF THE HUMAN BRAIN. By John B. Roberts, A. M., M. D., Professor of Anatomy and Surgery in the Philadelphia Polyclinic, Surgeon to St. Mary's Hospital. Philadelphia: P. Blackeston, Son & Co. 1885.

This monograph is divided into three chapters. Chapter I., Principles of cerebral surgery. Chapter II., Cerebral localization. Chapter III., Operative treatment of cerebral lesions. Far from being exhaustive, it presents in a limited space a most valuable collection of knowledge on this completely new branch of operative surgery. The book is written in a pleasing style, and the work of the publishers is neat and attractive.

